# Valorization and Mechanical Recycling of Heterogeneous Post-Consumer Polymer Waste through a Mechano-Chemical Process

**DOI:** 10.3390/polym13162783

**Published:** 2021-08-19

**Authors:** Roberta Capuano, Irene Bonadies, Rachele Castaldo, Mariacristina Cocca, Gennaro Gentile, Antonio Protopapa, Roberto Avolio, Maria Emanuela Errico

**Affiliations:** 1 Institute for Polymers, Composites and Biomaterials—IPCB, National Research Council of Italy (CNR), Via Campi Flegrei 34, 80078 Pozzuoli, Italy; roberta.capuano@ipcb.cnr.it (R.C.); irene.bonadies@ipcb.cnr.it (I.B.); rachele.castaldo@ipcb.cnr.it (R.C.); mariacristina.cocca@ipcb.cnr.it (M.C.); gennaro.gentile@ipcb.cnr.it (G.G.); mariaemanuela.errico@ipcb.cnr.it (M.E.E.); 2 Department of Mechanical and Industrial Engineering—DIMI, University of Brescia, Via Branze 38, 25121 Brescia, Italy; 3 Italian Consortium for the Collection and Recycling of Plastic Packages—COREPLA, Via del Vecchio Politecnico 3, 20121 Milano, Italy; protopapa@corepla.it

**Keywords:** polymer based post-consumer waste, mechano-chemical treatment, ball milling, mechanical recycling

## Abstract

In this paper, a sustainable strategy to valorize and recycle heterogeneous polymer-based post-consumer waste is proposed. This strategy is based on a high-energy mechano-chemical treatment and has been applied to a polyolefin-rich fraction, coded as FIL/S, deriving from household plastic waste collection. This processing, performed in a planetary ball mill, allowed us to obtain fine grinding and, consequently, to induce an intimate mixing of the different polymer fractions and contaminants composing the FIL/S, as demonstrated by SEM analysis. As a result, an improvement in the deformability of the treated material was obtained, recording values for elongation at the break which were two and half times higher than the neat FIL/S. Finally, the addition of small amounts of organic peroxide during mechano-chemical treatment was tested, determining a more homogeneous morphology and a further improvement in mechanical parameters.

## 1. Introduction

The versatility and performances of plastics have led to their use in virtually all of the major product categories, with applications spanning from household to aerospace. About 40% of the world consumption of plastics is in the packaging sector [1], which refers to food and beverages, pharmaceuticals, and personal and household products. It has been estimated that the value of the global plastic packaging market amounted to USD 348.08 billion in 2020 and it is expected to grow at a compound annual growth rate (CAGR) of 4.2% from 2021 to 2028 [2].

Plastic packaging is characterized by a quite short service life resulting in (a) a high rate of waste generation (the package is disposed of in a short time), and (b) high intrinsic value of the discarded materials (high quality raw materials are used for food contact, short service life produces relatively low degradation issues) [3,4].

Despite this, only a small part of post-consumer plastic packaging is actually recycled [5]. It is estimated that 95% of the material value of used plastic packaging, accounting to around USD 120 billion, is lost annually [6]. Then, in spite of important societal benefits deriving from the widespread use of plastics, the management of plastics at the end-of-life stage causes serious environmental and economic problems.

In the frame of a circular economy, a radical change of the waste concept is necessary: what was once considered as waste must become a valuable resource. This means addressing technological, economical and legislative challenges to move towards the maximization of secondary raw material recovery and recycling [5].

To develop efficient recycling strategies for polymer waste, some important issues must be addressed concerning the compositional and structural complexity of most plastic products; the contamination and the thermo-mechanical degradation affecting plastic during its life cycle; the limited efficiency of collection and sorting systems unable to accurately separate pure materials; the high compositional heterogeneity of the plastic waste stream, which depends on the geographical area as well as on the season [7,8]. Finally, it has to be considered that the low price of some virgin commodities does not encourage investment in large resources to improve recycling efficiency.

The compositional heterogeneity, caused by complex item composition (filler, additives, multilayered structures) and/or contamination by organic and inorganic substances during the life cycle and/or incomplete separation during the sorting procedure, represents a major technological challenge for recycling, in terms of the obtainable quality or properties of recycled materials [7].

It is well known that the realization of polymer-based multicomponent materials requires an effective strategy able to induce an intimate mixing between different polymer fractions, thus controlling morphology and properties of the blend. To this aim, several approaches have been reported in the literature, mainly involving the addition of compatibilizing agents and/or reactive additives such as anhydrides or peroxides during processing [9,10].

Polymeric compatibilizers are generally very effective, but their chemical structure needs to be carefully designed for a specific blend composition [11], making them unsuitable for the intrinsically heterogeneous and highly variable waste plastic mixtures. On the other hand, the addition of reactive substances during processing ensures greater flexibility and lower cost but, at the same time, could cause material degradation as well as the formation of extensive crosslinks, which makes the final properties very sensitive to processing conditions [12]. Therefore, to summarize, in order to maximize the recovery of secondary raw material from plastic waste, it is very important to define versatile, eco-friendly and cost-effective recycling approaches. 

In this paper, a strategy based on a high-energy mechanical treatment to valorize and recycle polyolefin-rich heterogeneous plastic waste is proposed. In particular, this mechano-chemical treatment was performed on a small-sized film fraction rich in polyolefins, named FIL/S, deriving from household collection and provided by COREPLA, by means of a planetary ball mill (BM).

This technology is traditionally used in the field of ceramics and metals to obtain a fine grinding and to produce new alloys and metastable compounds. Recently, it has been extended to polymeric systems as a solid-state strategy able to induce morphological and structural modifications [13,14], to enhance the dispersion of various nanofillers in composites [15] and as a tool to realize recycled polymeric materials with improved properties [16,17,18].

On this basis, FIL/S was processed in a BM to investigate the effects induced by the intense mechanical stresses on morphology and properties. It is important to underline that the mechano-chemical treatment has been performed at room temperature and in absence of solvents, thus responding to the requirements of eco-friendly processes. Moreover, the possibility to promote the compatibilization between different fractions by adding a small amount of an organic peroxide during the ball milling treatment has been explored. 

Before processing, FIL/S was characterized performing spectroscopic (solid state NMR and FTIR) analyses, to evaluate its composition [19] and define treatment conditions. Processed materials were analyzed through morphological and mechanical analyses, assessing processing–structure–properties relationships.

## 2. Materials and Methods

### 2.1. Materials

FIL/S, post-consumer plastic films of a small size, was kindly supplied by COREPLA (Italian Consortium for the Collection and Recycling of Plastic packages, Milano, Italy). This material is one of the fractions derived from the sorting process of household plastic waste; it contains films smaller than an A3 sheet (approximately 30 × 40 cm), recovered by air aspiration during the waste sorting process and shredded to few-centimeter fragments.

Di-benzoyl peroxide (BPO), Fluka, reagent grade, was used without further purification.

Low-density polyethylene (Lupolen 2426 H, density 0.925 g/cm^3^, MFR 1.9 g/10 min) was kindly supplied by COREPLA and used as a reference material.

### 2.2. Processing of FIL/S

FIL/S material was ground in a SM100 rotary knife mill (Retsch GmbH, Haan, Germany), using a bottom sieve with 4 mm openings. 

Ground FIL/S was processed in a PM100 planetary ball mill (Retsch GmbH, Haan, Germany), using either 125 or 500 mL steel grinding bowl and 10 or 20 mm steel balls. The ball/sample weight ratio was set at 10/1. Different bowl rotation speed and grinding time were tested, as specified in the Section 3, ranging from 4 to 10 h and from 400 to 600 rpm.

Moreover, ground FIL/S was processed in combination with 0.5 and 1 wt% of BPO: pristine ground FIL/S was ball milled for 2 h to obtain a fine powder with high surface area, then the peroxide was added and the BM process continued for further 2 h.

Ball-milled materials were processed in a benchtop twin-screw extruder (Haake Minilab, Haake, Germany) operated in continuous mode, at a screw rotation speed of 60 rpm and a barrel temperature of 180 °C. Then, materials were pelletized and successively compression molded in a heated press at 190 °C and 50 bar obtaining 1.5 mm-thick sheets to be used for subsequent analysis. 

### 2.3. Techniques

Infrared spectra were recorded by means of a Spectrum 100 FTIR spectrometer (PerkinElmer, Waltham, MA, USA), equipped with an attenuated total reflectance accessory (ATR). The scanned wavenumber range was 4000–400 cm^−1^. All spectra were recorded with a resolution of 4 cm^−1^, and 16 scans were averaged for each sample.

Solid-state ^13^C Magic Angle Spinning (MAS) Nuclear Magnetic Resonance (NMR) spectra were collected on a Bruker Avance II 400 spectrometer (Bruker Biospin, Billerica, MA, USA) operating at a static field of 9.4 T, equipped with a 4 mm MAS probe. Ground FIL/S samples were packed into 4 mm zirconia rotors sealed with Kel-F caps and spun at 5 kHz. Cross-polarization (CP) spectra were recorded with a relaxation delay of 5 s and a contact time of 2 ms under high-power proton decoupling. Spectra were referenced to external adamantane (CH_2_ signal at 38.48 ppm downfield of tetramethylsilane (TMS), set at 0.0 ppm).

Tensile tests were performed on dumb-bell specimens (6 mm^2^ cross section, 1.5 mm thickness, 26 mm gauge length) at a crosshead speed of 10 mm/min by using an Instron 5564 testing machine (ITW Inc., Glenview, IL, USA). Young’s modulus (E), peak stress (σ), and elongation at break (ε) were calculated as average values over at least 6 tested samples.

Scanning electron microscopy (SEM) was carried out on a Quanta 200 FEG microscope (FEI, Hillsboro, OR, USA) working in high vacuum mode with an acceleration voltage ranging from 10 to 30 kV and using a secondary electron detector. Before SEM observations, cryofractured surfaces were sputter coated with an Au/Pd alloy by means of an Emitech K575X sputtering device.

Image analysis was carried out on SEM micrographs to obtain quantitative geometrical information on the dispersed phase, by means of the ImageJ software package. Dispersed phase inclusions were manually identified and fitted to ellipses (Figure S1 in the Supplementary Materials).

## 3. Results

### 3.1. Analysis of FIL/S

Spectroscopic analyses were performed on the FIL/S to better clarify its composition as well as any degradation phenomena affecting the FIL/S polymer fractions as a result of the life cycle.

**Figure 1:**
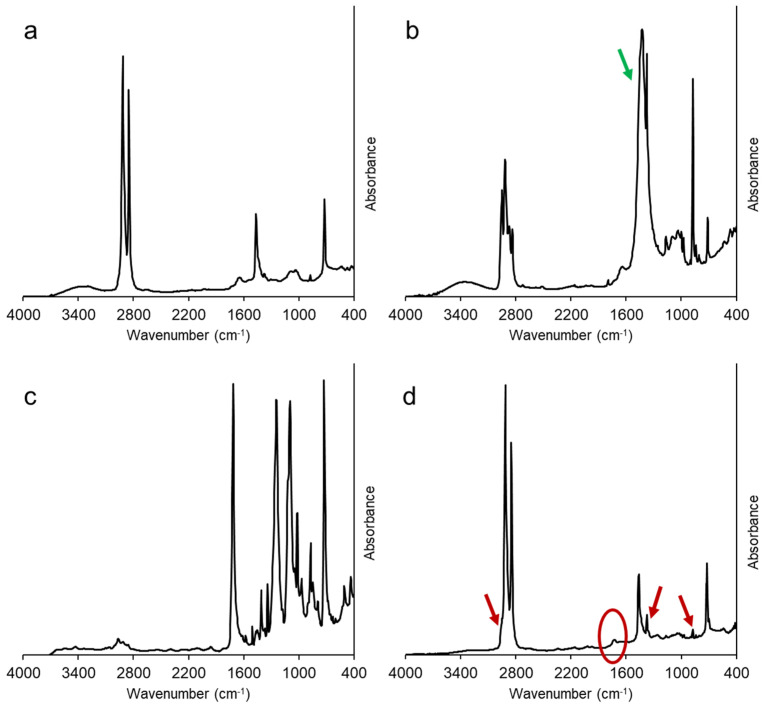
ATR-FTIR spectra of selected FIL/S film fragments (**a**–**c**) and of a film obtained after melt mixing and molding (**d**). In panel b, the signal attributed to calcium carbonate is indicated by a green arrow. In panel d, red arrows indicate polypropylene signals, while the small carbonyl peak is highlighted by a red ellipse.

Considering the high heterogeneity of the provided material, ATR-FTIR spectroscopy was performed on several different film fragments, with some examples reported in Figure 1.

The majority of films analyzed showed the typical absorption of polyethylene (PE), as reported in Figure 1a, with strong peaks at 2916, 2850, 1470, and 720 cm^−1^ due to CH_2_ asymmetric and symmetric stretching, bending and rocking, respectively. The presence of some weaker bands in the spectrum can be related to additives (stabilizers and pigments) and surface contamination. The spectra of a minor family of film fragments, as in Figure 1b, reveal the presence of polypropylene (PP) with the typical, composite absorption bands in the range 2980–2830 cm^−1^—in some cases, filled with inorganic additives, such as calcium carbonate, whose adsorption is indicated by the green arrow [20]. The presence of polyethylene terephthalate (PET) films, with main absorptions of the ester group (carbonyl at 1715 cm^−1^, C-O at 1240 and 1095 cm^−1^), phenyl ring (1408 and 1340 cm^−1^) [21], often laminated with PE or PP, was evident in some samples (Figure 1c). An “averaged” composition can be observed in the spectrum reported in Figure 1d, recorded after melt processing and molding: the main features of PE can be easily identified, with much less intense peaks attributed to PP (875, 1375 cm^−1^ and the shoulder at 2950 cm^−1^, indicated by red arrows). A weak absorption in the carbonyl region (1720 cm^−1^) can also be observed, which could be attributed to organic contaminants (e.g., PET, as observed in Figure 1c) and to a possible limited oxidative degradation of the polyolefin fractions [22]. 

**Figure 2:**
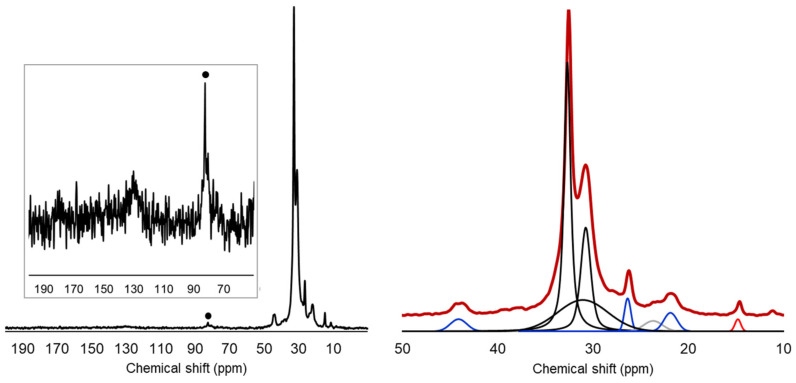
^13^C solid-state NMR spectrum of FIL/S, left, with insert showing a magnification of the aromatic/carbonyl region. On the right, spectral deconvolution of the region containing the main signals of PE and PP. Spinning sidebands are marked by a dot.

^13^C solid-state NMR was also performed on finely ground FIL/S samples, as reported in Figure 2, to elucidate and quantitatively define the composition of the FIL/S mixture.

Analyzing the ^13^C spectrum, the main resonances are found in the 10–50 ppm region and are assigned to PE (intense peaks centered at 30.8 and 32.6 ppm) and PP (signals at about 22, 26 and 44 ppm) moieties. The peak observed at 15 ppm was assigned to methyl groups of LDPE/LLDPE chain branches [23]. In the low-field section of the spectrum, reported in the insert at high magnification, some residual signals of unsaturated carbons and carbonyls (about 130 and 175 ppm) can be observed, while a signal around 70 ppm is partially masked by the intense spinning sideband centered at about 75 ppm (marked by a dot in Figure 2). These signals are compatible with the presence of PET [24], in agreement with FTIR analysis. 

Through a spectral deconvolution procedure, the peaks relative to the different components were isolated and the respective areas were calculated. The result of deconvolution is reported graphically in the right panel of Figure 2: peaks assigned to PE, PP and methyl terminals of PE branches are black, blue and red, respectively. It is to be noted that PE main chain at the solid state shows multiple resonances, due to the coexistence of crystalline and amorphous domains. Comparing the areas calculated, the content of PP was estimated at 12 wt%. Moreover, assuming that the number of chain branches (NB) is 20 for every 1000 CH_2_ groups in the main chain, a reasonable estimation for LLDPE [25], from the area of the peak at 15 ppm it was evaluated that about 65% of PE in the FIL/S mixture is branched.

In summary, spectroscopic analyses clarified that the FIL/S mixture was mainly composed by polyethylene, of which at least 65 wt% is branched, in addition to a moderate (12 wt%) amount of PP, traces of inorganic fillers and polymeric contaminations (essentially PET fragments). 

### 3.2. BM Treatment, Processing and Testing

The findings reported confirm the compositional complexity of the FIL/S mixture, as PE families with different chain structures are not easily processed together, and are generally not miscible with PP. To set up a versatile processing strategy able to avoid phase separation and to allow the valorization and the recycling of the FIL/S, our approach was based on high-energy mechanical treatment [17]. The material, previously grounded as reported in the experimental part, was processed in a planetary ball mill, consisting of a steel milling jar containing steel balls and subjected to a planetary-like rotation-revolution motion. The balls accelerated by the fast rotation of the jar, generates strong local shear and compressive stresses on the processed materials.

Processing conditions were optimized changing jar and ball size, ball-to-material weight ratio, rotation rate and processing time. As described in Section 2, two general BM conditions were selected: a “high energy” setup, obtained using 20 mm steel balls and a 500 mL jar, and a “low energy” setup based on 125 mL jars and 10 mm balls. Larger balls in fact result in higher-impact energy, and larger jars due to their larger diameter increase the acceleration of balls. For the high-energy conditions, rotation speed was limited to 400 rpm as any further increase led to overheating with a partial melting of the materials, while using 125 mL jars allowed rotation speed up to 600 rpm. Ball-milled samples, reduced to a fine powder, were then melt processed and compression molded to 1.5 mm-thick sheets and characterized, performing tensile tests and morphological analyses. For comparison, untreated FIL/S and a commercial neat LDPE were also characterized. In Table 1, the processing conditions, the relative code of the processed sample and the main mechanical parameters are resumed. The ball-milled samples have been identified with A × B type codes where A represents the duration in hours of the treatment and B represents the rotation rate of the ball mill.

The untreated FIL/S shows a low elastic modulus and low strength, comparable to those of a low-density polyethylene (LDPE) and in line with compositional analysis that identified branched PE as the main component of the mixture. However, a significantly lower value of the elongation at break than that of commercial LDPE was also recorded. The low evidence of signals relative to oxidized groups in the spectroscopic analyses allows to exclude thermo-oxidative degradation of the polymers as the cause of the low elongation observed. Then, this behavior could be attributed to the heterogeneity of the mixture. The presence of different immiscible polymers in a bulk mainly composed by LDPE causes an embrittlement of the material because these fractions act as defects generating a premature failure of the sample [26]. Observing data of treated materials, the ball milling does not affect the tensile modulus and the stress at break values, which are comparable to those of untreated FIL/S. On the contrary, the mechano-chemical treatment induces an improvement as concerning the deformability of the samples, in particular the elongation at break value recorded on the of 8 × 600 and 4 × 400 samples is two and half times higher than the neat FIL/S.

**Figure 3:**
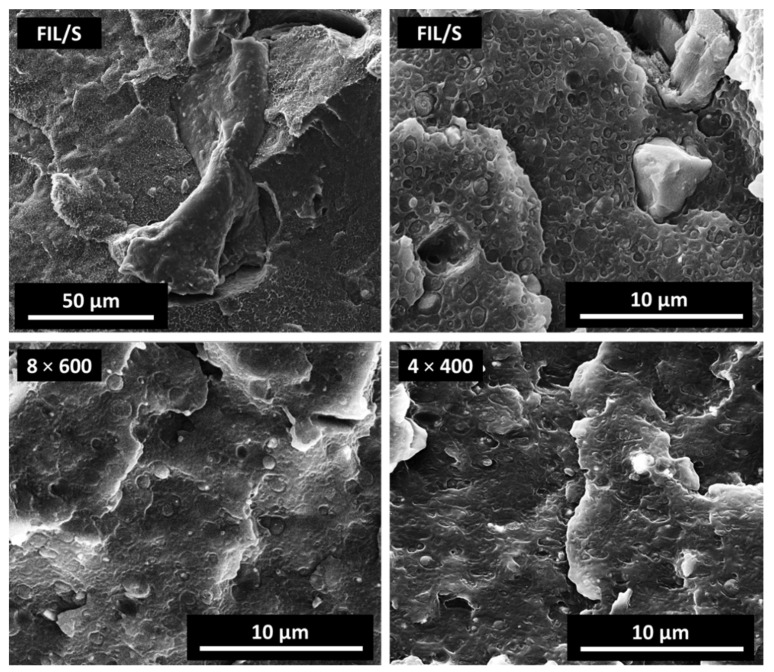
SEM micrographs of cryo-fractured surfaces of FIL/S, 8 × 600 and 4 × 400 samples.

Morphological analysis was performed on cryogenically fractured surface of the untreated and BM treated samples, to further investigate the effects of BM treatments: SEM micrographs of untreated FIL/S and of samples 8 × 600 and 4 × 400 are shown in Figure 3.

Comparing the morphology of the different materials, ball milling revealed a double effect on the structure of treated samples. First, large (few µm to tens of µm) inclusions with irregular shape, frequently observed in neat FIL/S, are practically absent in BM treated materials, evidencing a very effective homogenization induced by the milling. Such inclusions appear completely debonded from the FIL/S matrix and are the main responsible for the low elongation shown by FIL/S, representing defects and failure-starting points [27]. Large, film-like inclusions such as the one observed in the first panel of Figure 3 may be attributed to polymeric contaminants such as PET, not melted during the processing. As a second finding, globular inclusions of micrometric and submicrometric size, observed in large numbers in FIL/S (affected, again, by evident debonding and pull-out due to the low interfacial adhesion), are less evident in BM treated samples where they appear homogeneously dispersed and partially covered/anchored to the polymer bulk. Image analysis carried out on SEM micrographs showed that the area occupied by the dispersed phase (approximated by 2D elliptical shapes, see Supplementary Information) is much larger in FIL/S, than in the BM-treated samples. As shown in Table 2, the dispersed phase represents almost 20% of the fracture surface in FIL/S and is reduced to 7.4 and 4.6% in 8 × 600 and 4 × 400 samples, respectively. These observations underline a strong beneficial effect of the intimate mixing of the different polymeric phases induced by BM also at a micrometric level [17].

In summary, SEM analysis confirms the effect of the BM pretreatment on the morphology of the prepared materials, which is the substantial size reduction of dispersed inclusions, thus resulting in the intimate mixing of different components and consequently in the improved homogeneity of the mixture. These effects justify lower occurrence of debonding phenomena and determine the enhancement of the elongation at break observed in mechanical tests for samples 8 × 600 and 4 × 400. 

Moreover, in addition to the size reduction of inclusions, the BM processing could also promote, through mechanical stresses and local temperature increase produced by high-energy impacts, the formation of reactive radical species, with the in situ generation of graft copolymers able to actively compatibilize polymer blends [28]. These kinds of reactions could be very useful to achieve a versatile, non-specific compatibilization of polymer mixtures, using a simple and solvent-free process.

### 3.3. BM Treatment Coupled to the Presence of Peroxide

To explore the possible role of radical species formation and their reactions at the solid state, moderate amounts (0.5 and 1 wt%) of benzoyl peroxide (BPO) were added during the ball milling process. The BM treatment parameters granting the best properties/BM time balance (4 × 400) were selected for such test. BPO was added after 2 h of milling, to ensure a sufficient grinding and, thus, a high available surface area; the treatment was then continued for 2 further hours.

After processing and compression molding, tensile tests were performed. The results of mechanical analysis are reported in Table 3 and compared with values of the unprocessed FIL/S and 4 × 400 materials.

In the presence of BPO, a significant increase in elastic modulus was recorded, correlated to the amount of peroxide. These data suggest that BPO is likely to induce some level of crosslinking in the polyethylene matrix, responsible for the increased stiffness. A very light degree of crosslinking can be hypothesized, as the materials were processed in the extruder and compression molded without any evidence of gels or obstructions to viscous flow. Notwithstanding the increased stiffness, BPO-containing materials showed a higher ultimate elongation with respect to FIL/S and, at 1 wt% of BPO, even higher than the 4 × 400 sample. The effect of peroxides on single polyolefins and on their blends has been widely investigated, reporting beneficial effects on the compatibility of PE-PP blends [29] but also a crosslinking effect on the PE fraction [30]. The deformability of materials is strongly dependent on composition, generally decreasing with increasing peroxide content [31,32].

**Figure 4:**
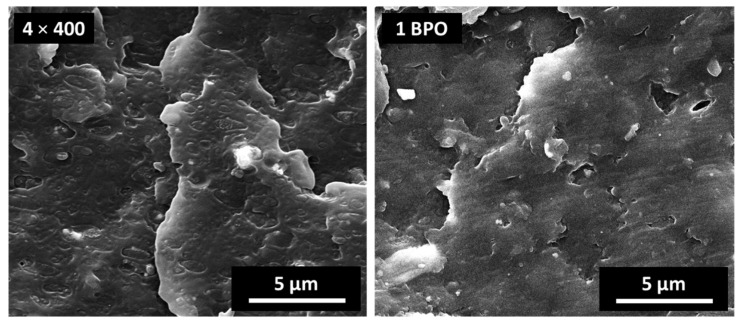
SEM micrographs of cryo-fractured surfaces of 4 × 400 and 1 BPO (2 + 2) × 400 samples.

Interestingly, it has been reported that the use of peroxides at low temperature, in solution [30] or at the solid state [33], can largely prevent crosslink/degradation of polyolefins. We can, thus, conclude that the addition of BPO during the BM process, at moderate temperatures (maximum T recorded is 80 °C), followed by the extrusion process of the blend, has a lower adverse effect on the polymer structure in comparison to the direct addition of BPO during melt processing at temperatures above 180 °C (attempts to directly process FIL/S with BPO in our benchtop extruder led to unstable flow and highly degraded materials). The higher elongation showed at higher BPO content suggests a synergistic effect of BM treatment and peroxide action on the structure of the final materials, which will require further studies to be fully elucidated. SEM analysis of the best performing material, 1 BPO (2 + 2) × 400, is shown in Figure 4 in comparison with 4 × 400.

Spherical, immiscible inclusions are much less evident in the sample processed with BPO, thus suggesting the achievement of an effective compatibilization through the reactive BM treatment. Although a mixing of the heterogeneous polymer mixture at molecular level is unlikely, the better dispersion and stronger interfacial adhesion induced by the processing reduce interface failure during cryo-fracturation, resulting in smoother surfaces. 

## 4. Conclusions

In this paper, a strategy based on high-energy mechanical treatment was investigated to valorize and recycle polyethylene-rich heterogeneous post-consumer mixture. This strategy allows us to induce the fine grinding of different polymeric fractions and contaminants, thus promoting an intimate mixing between different components. As a result, an improvement in mixture morphology and a higher deformability were obtained. Then, the addition of small amounts of benzoyl peroxide during the ball-milling process was also explored to promote radical formation. The low temperature of the process reduced the adverse effects of the peroxide on polymers, granting higher stiffness while retaining a significant elongation at break, phenomena ascribable to the formation of very light crosslinking.

This technology can be considered an advancement towards sustainability, considering that the treatments were carried out in absence of solvent and at room temperature and no further purification/refinement steps were needed.

## Figures and Tables

**Table 1:** Codes, BM conditions and mechanical parameters of the recycled materials.

BM Geometry (Jar Vol. Ball φ)	BM Conditions (Time, Speed)	Code	E (MPa)	σ (MPa)	ε (%)
-	-	LDPE	300 ± 30	12.9 ± 0.5	450 ± 8
	-	FIL/S	348 ± 6	11.0 ± 0.7	20 ± 8
125 mL jar 10 mm balls (Low Energy)	4 h, 600 rpm	4 × 600	330 ± 10	11.1 ± 0.3	20 ± 10
	8 h, 600 rpm	8 × 600	317 ± 5	10.5 ± 0.1	60 ± 10
	10 h, 600 rpm	10 × 600	340 ± 20	11.2 ± 0.1	30 ± 10
500 mL jar 20 mm balls (High Energy)	4 h, 400 rpm	4 × 400	320 ± 10	10.9 ± 0.1	54 ± 3
	8 h, 400 rpm	8 × 400	321 ± 6	11.1 ± 0.2	47 ± 8

**Table 2:** Surface fraction attributed to the dispersed phase obtained by image analysis of SEM micrographs.

Sample	Dispersed Phase Area (%)
FIL/S	19.8
8 × 600	7.4
4 × 400	4.6

**Table 3:** Codes and mechanical parameters of the materials treated with BPO, as compared to neat FIL/S and 4 × 400 samples.

Additive	Code	E (MPa)	σ (MPa)	ε (%)
-	FIL/S	348 ± 6	11.0 ± 0.7	20 ± 8
-	4 × 400	320 ± 10	10.9 ± 0.1	54 ± 3
0.5 wt% BPO	0.5 BPO (2+2) × 400	444 ± 6	10.5 ± 0.3	40 ± 10
1 wt% BPO	1 BPO (2+2) × 400	520 ± 20	11.5 ± 0.2	60 ± 10

## Data Availability

Not applicable.

## Supplementary Materials

The following are available online at https://www.mdpi.com/article/10.3390/polym13162783/s1, Figure S1: Areas selected for image analysis with contrast enhancement; Figure S2: Dispersed phase inclusions identified and fitted to ellipses; Table S1: Parameters calculated from image analysis on FIL/S.
