# Supplementary material for: Valorization and Mechanical Recycling of Heterogeneous Post-Consumer Polymer Waste through a Mechano-Chemical Process
Source: Polymers (Basel). 2021 Aug 19;13(16):2783. doi: 10.3390/polym13162783 (PMC8399838; doi:10.3390/polym13162783)
Supplement: Supplementary file 1 [file polymers-13-02783-s001.zip › polymers-1331068-supplementary.pdf]

Supplementary material

Valorization and mechanical recycling of heterogeneous post-consumer polymer waste through a mechano-chemical process

Roberta Capuano[1,2,†], Irene Bonadies[1,†], Rachele Castaldo[1], Mariacristina Cocca[1], Gennaro Gentile[1], Antonio Protopapa[3], Roberto Avolio[1,*], Maria Emanuela Errico[1]

[1] Institute for Polymers, Composites and Biomaterials-IPCB, National Research Council (CNR), Via Campi Flegrei, 34 - 80078 Pozzuoli (Na), Italy; roberta.capuano@ipcb.cnr.it (R.C1.); irene.bonadies@ipcb.cnr.it (I.B.); rachele.castaldo@ipcb..cnr.it (R.C2.); mariacristina.cocca@ipcb.cnr.it (M.C.); gennaro.gentile@ipcb.cnr.it (G.G.); mariaemanuela.errico@ipcb.cnr.it (M.E.E.)
[2] Department of Mechanical and Industrial Engineering - DIMI, University of Brescia, via Branze, 38 - 25121 Brescia, Italy
[3] Italian Consortium for the Collection and Recycling of Plastic Packages - COREPLA, Via del Vecchio Politecnico, 3 - 20121 Milano, Italy; protopapa@corepla.it (A.P.)
* Correspondence: roberto.avolio@ipcb.cnr.it (R.A.)
† These authors contributed equally to this work

1. Image analysis

Image analysis was carried out on SEM micrographs of cryo-fractured surfaces, to obtain quantitative geometrical information on the dispersed phase.

The ImageJ software package was used for the analysis. Contrast and brightness of the micrographs were adjusted to evidence any discontinuity in the surface, then the dispersed phase was identified and inclusions were manually fitted to ellipses. For neat FIL/S, the area of the micrograph containing large (> 5 μm) inclusions of irregular shape was excluded from the analysis.

In Figures S1 and S2, the analyzed areas and the results of dispersed phase identification are shown, respectively.

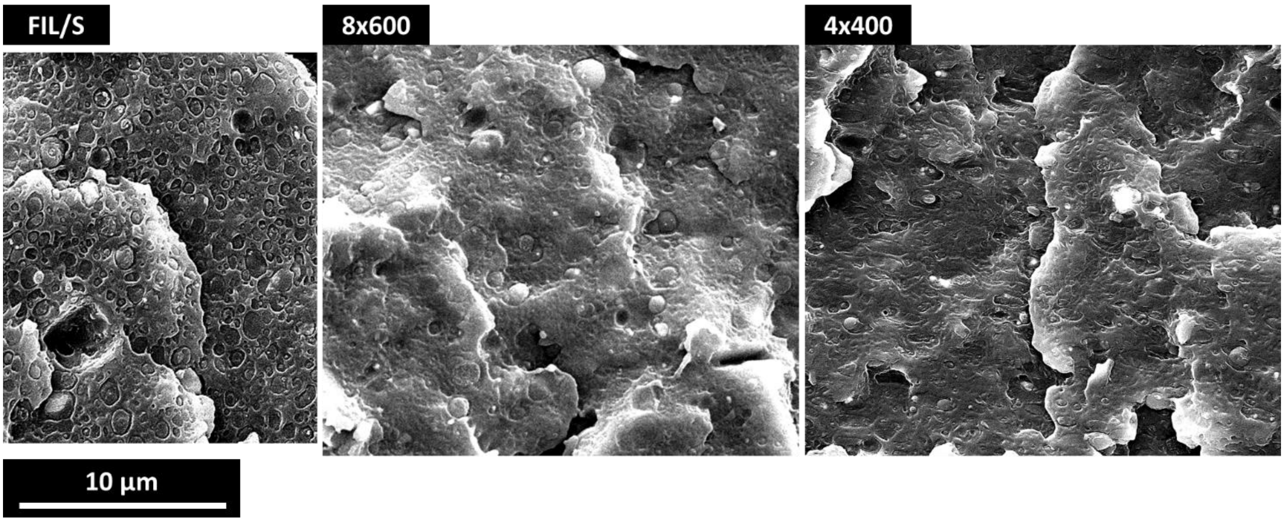

Figure S1. Areas selected for image analysis with contrast enhancement

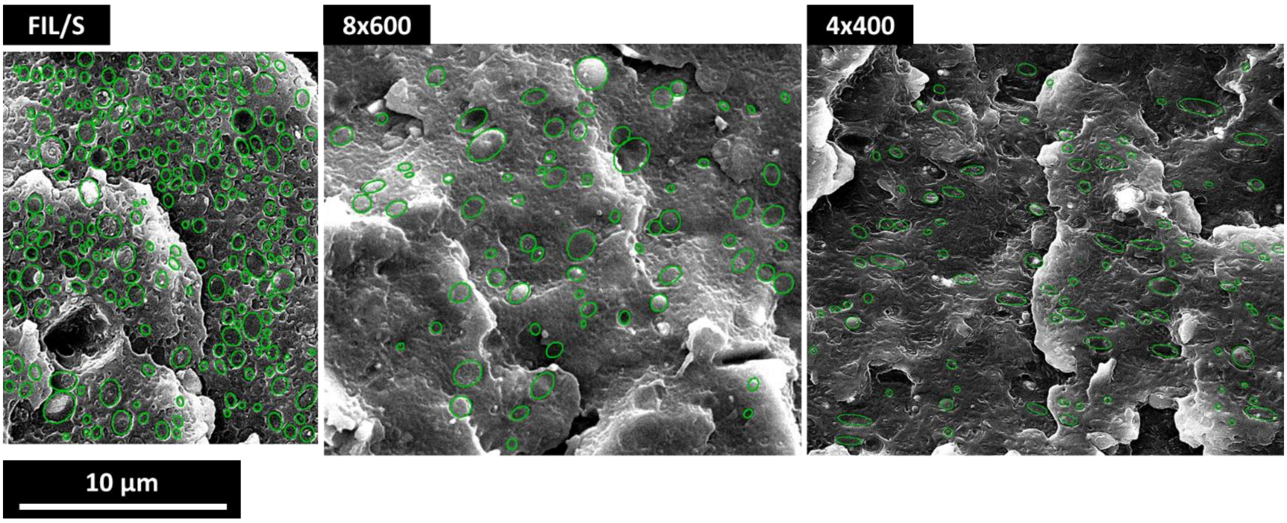

Figure S2. Dispersed phase inclusions identified and fitted to ellipses

Geometrical parameters of the identified inclusions are reported in Tables S1, S2 and S3 for FIL/S, 8x600 and 4x400 samples, respectively. Circularity is calculated as 4π(area/perimeter^2).

Table S1. Parameters calculated from image analysis on FIL/S

n	Area	Major	Minor	Angle	Circularity
1	0.056	0.316	0.226	90	0.932
2	0.197	0.575	0.436	90	0.952
3	0.229	0.653	0.447	90	0.954
4	0.253	0.617	0.522	90	1
5	0.181	0.514	0.449	90	1
6	0.581	0.884	0.836	90	1
7	0.153	0.486	0.402	90	1
8	0.39	0.705	0.705	0	1
9	0.208	0.514	0.514	0	1
10	0.072	0.395	0.232	90	0.912
11	0.33	0.721	0.583	90	0.969
12	0.288	0.634	0.578	90	1
13	0.34	0.689	0.627	0	1
14	0.186	0.55	0.432	90	0.972
15	0.414	0.849	0.621	90	0.979
16	0.119	0.465	0.324	0	0.921
17	0.181	0.577	0.4	90	0.934
18	0.304	0.663	0.584	90	1
19	0.886	1.062	1.062	0	1
20	0.645	1.022	0.803	90	0.979
21	1.435	1.425	1.281	90	1
22	0.218	0.586	0.475	0	0.988
23	0.068	0.328	0.264	0	1
24	0.085	0.376	0.289	90	1
25	0.068	0.328	0.264	90	1

26	0.374	0.768	0.62	90	0.986
27	0.474	0.87	0.694	90	0.974
28	0.967	1.13	1.089	90	0.994
29	0.414	0.849	0.621	90	0.979
30	0.769	1.067	0.917	90	1
31	0.434	0.843	0.656	90	0.98
32	0.069	0.297	0.297	0	1
33	0.069	0.297	0.297	0	1
34	0.314	0.723	0.553	90	0.971
35	0.181	0.577	0.4	90	0.934
36	0.301	0.802	0.478	90	0.909
37	0.507	0.961	0.672	55.104	0.96
38	0.341	0.847	0.513	121.582	0.906
39	0.304	0.791	0.489	120.69	0.911
40	0.574	1.106	0.661	96.313	0.91
41	0.413	0.78	0.674	103.423	0.982
42	0.37	0.778	0.606	82.876	0.976
43	0.117	0.438	0.341	122.882	0.987
44	0.226	0.6	0.481	100.618	0.99
45	0.127	0.555	0.29	78.175	0.85
46	0.787	1.156	0.867	88.179	0.961
47	0.282	0.679	0.53	130.196	0.99
48	0.103	0.403	0.324	137.912	0.951
49	0.148	0.483	0.39	92.85	0.966
50	0.763	0.986	0.986	0	1
51	0.442	0.844	0.667	92.375	0.981
52	0.232	0.602	0.49	132.701	0.985
53	0.053	0.293	0.232	90	0.977
54	0.162	0.492	0.421	114.612	1
55	0.1	0.378	0.337	107.89	0.969
56	0.278	0.633	0.56	90	0.99
57	0.252	0.613	0.523	88.175	0.999
58	0.196	0.533	0.468	125.013	1
59	0.162	0.491	0.421	120.474	1
60	0.201	0.546	0.469	116.996	0.976
61	0.18	0.619	0.37	42.765	0.91
62	0.218	0.619	0.449	100.418	0.968
63	0.281	0.632	0.566	90	0.984
64	0.288	0.71	0.516	86.518	0.952
65	0.209	0.604	0.441	94.817	0.964
66	0.095	0.348	0.346	45	0.978
67	0.217	0.581	0.476	77.776	0.996
68	0.196	0.552	0.451	90	1
69	0.124	0.438	0.36	135	0.993
70	0.047	0.283	0.21	59.093	0.885
71	0.292	0.672	0.553	69.054	0.987

72	0.213	0.655	0.414	98.749	0.92
73	0.085	0.402	0.27	116.138	0.928
74	0.117	0.474	0.315	138.18	0.926
75	0.104	0.445	0.297	82.58	0.955
76	0.103	0.442	0.295	136.473	0.911
77	0.174	0.574	0.387	132.295	0.934
78	0.144	0.522	0.351	133.99	0.931
79	0.055	0.32	0.217	121.148	0.972
80	0.137	0.52	0.336	121.186	0.967
81	0.048	0.309	0.197	138.144	0.985
82	0.08	0.387	0.263	126.77	0.93
83	0.079	0.377	0.265	126.141	0.946
84	0.043	0.285	0.19	135	0.959
85	0.075	0.382	0.248	111.599	0.875
86	0.31	0.656	0.602	131.324	0.986
87	0.286	0.75	0.486	84.923	0.926
88	0.493	1.044	0.601	105.487	0.899
89	0.087	0.432	0.255	130.098	0.885
90	0.131	0.414	0.402	134.984	0.972
91	0.063	0.393	0.203	59.98	0.873
92	0.089	0.443	0.257	83.866	0.899
93	0.166	0.594	0.357	58.955	0.914
94	0.089	0.446	0.255	56.932	0.924
95	0.099	0.467	0.269	64.455	0.923
96	1.067	1.432	0.948	116.563	0.944
97	0.429	0.79	0.691	102.636	0.991
98	0.165	0.604	0.348	86.83	0.876
99	0.067	0.38	0.223	113.461	0.892
100	0.079	0.377	0.265	97.644	0.931
101	0.252	0.683	0.469	99.057	0.952
102	0.718	1.144	0.799	94.238	0.95
103	0.882	1.269	0.885	90	0.949
104	0.286	0.714	0.51	90.303	0.943
105	0.077	0.386	0.255	107.338	0.959
106	0.08	0.383	0.265	96.055	0.927
107	0.057	0.356	0.205	119.82	0.911
108	0.161	0.5	0.411	82.969	0.962
109	0.586	0.97	0.769	139.285	0.982
110	0.205	0.581	0.45	80.212	0.985
111	0.376	0.781	0.613	70.667	0.974
112	0.056	0.299	0.238	123.951	0.999
113	0.165	0.52	0.405	105.185	0.986
114	0.097	0.397	0.312	120.878	0.955
115	0.105	0.406	0.33	96.987	0.996
116	0.06	0.318	0.24	48.392	0.978
117	0.189	0.57	0.422	99.209	0.965

118	0.048	0.279	0.219	136.651	0.989
119	0.036	0.249	0.184	76.952	0.978
120	0.781	1.242	0.8	57.327	0.936
121	0.234	0.682	0.437	101.923	0.954
122	0.22	0.661	0.423	121.23	0.947
123	0.635	1.04	0.778	52.726	0.972
124	0.242	0.634	0.487	75.549	0.988
125	0.482	0.906	0.677	48.813	0.971
126	0.262	0.631	0.529	97.343	0.991
127	0.382	0.754	0.645	67.464	0.993
128	0.081	0.358	0.289	110.221	1
129	0.254	0.661	0.49	106.713	0.973
130	0.237	0.641	0.471	122.566	0.953
131	0.892	1.252	0.907	77.557	0.96
132	0.928	1.291	0.916	98.447	0.958
133	0.477	0.845	0.718	82.504	0.993
134	0.348	0.861	0.514	45.002	0.883
135	0.213	0.678	0.4	39.782	0.92
136	0.284	0.78	0.463	54.161	0.901
137	0.381	0.908	0.534	138.407	0.907
138	0.06	0.358	0.213	132.141	0.894
139	0.041	0.299	0.176	63.569	0.885
140	0.087	0.427	0.258	40.845	0.877
141	0.178	0.622	0.365	128.076	0.896
142	0.077	0.407	0.242	110.17	0.899
143	0.599	0.976	0.782	98.985	0.985
144	0.246	0.638	0.492	21.199	0.966
145	0.184	0.557	0.42	125.107	0.972
146	0.121	0.408	0.378	172.407	0.991
147	0.324	0.707	0.583	71.623	0.978
148	0.084	0.336	0.318	121.409	1
149	0.245	0.562	0.556	136.866	1
150	0.868	1.208	0.916	63.231	0.967
151	0.168	0.535	0.399	97.09	0.977
152	0.481	0.905	0.677	83.244	0.971
153	0.381	0.869	0.558	55.146	0.921
154	0.306	0.786	0.496	112.465	0.92
155	0.293	0.75	0.497	96.967	0.917
156	0.2	0.628	0.405	70.025	0.92
157	1.73	1.693	1.301	43.955	0.975
158	0.916	1.278	0.913	80.217	0.963
159	0.923	1.317	0.893	4.677	0.938
160	0.3	0.752	0.507	58.615	0.954
161	0.159	0.496	0.407	100.299	0.986
162	0.12	0.435	0.351	40.392	0.997
163	0.108	0.413	0.332	72.059	1

164	0.075	0.33	0.288	134.998	0.971
165	0.091	0.381	0.302	103.252	0.988
166	0.093	0.387	0.307	97.018	0.991
167	0.065	0.323	0.258	109.375	1
168	0.069	0.414	0.213	118.2	0.838
169	0.987	1.173	1.071	139.214	0.994
170	0.116	0.448	0.329	84.871	0.963
171	0.043	0.274	0.198	120.305	1
172	0.037	0.259	0.184	130.667	0.866
173	0.039	0.258	0.191	135	0.913
174	0.096	0.427	0.286	111.982	0.926
175	0.176	0.554	0.404	132.448	0.947
176	0.107	0.451	0.301	113.818	0.953
177	0.365	0.815	0.57	98.873	0.961
178	0.127	0.478	0.337	119.76	0.947
179	0.049	0.291	0.216	112.224	0.987
180	0.099	0.428	0.293	70.199	0.94
181	0.189	0.577	0.417	129.274	0.932
182	0.178	0.575	0.395	120.317	0.947
183	0.06	0.326	0.234	137.765	1
184	0.389	0.84	0.589	84.187	0.949
185	0.041	0.261	0.201	121.037	0.99
186	0.093	0.402	0.296	76.43	0.937
187	0.028	0.222	0.16	118.619	1
188	0.208	0.687	0.385	152.483	0.877
189	0.159	0.495	0.407	125.049	1
190	0.047	0.283	0.21	120.907	0.906
191	0.031	0.24	0.163	33.306	0.977
192	0.076	0.366	0.264	128.069	0.975
193	0.075	0.357	0.266	129.076	0.958
194	0.16	0.524	0.389	55.303	0.969
195	0.221	0.618	0.455	64.257	0.965
196	0.107	0.442	0.307	68.313	0.955
197	0.412	0.879	0.596	111.796	0.941
198	0.089	0.345	0.329	45.007	0.995
199	0.822	1.024	1.022	78.309	0.997
200	0.489	0.789	0.789	47.749	0.991
201	0.105	0.368	0.364	135	1
202	0.125	0.417	0.383	90	1
203	0.029	0.203	0.184	134.999	0.974
204	0.145	0.491	0.377	70.326	0.95
205	0.141	0.492	0.365	127.37	0.948
206	0.067	0.335	0.253	93.648	0.965
207	0.052	0.312	0.212	114.675	0.944
208	0.155	0.526	0.374	109.688	0.95
209	0.067	0.346	0.245	113.981	0.927

210	0.043	0.233	0.233	0	1
211	0.068	0.301	0.288	119.488	0.981
212	0.101	0.371	0.347	90	0.996
213	0.053	0.266	0.255	54.277	1
214	0.112	0.43	0.331	126.089	0.979
215	0.067	0.32	0.265	49.429	1
216	0.099	0.44	0.285	50.16	0.935
217	0.663	1.387	0.609	116.759	0.787
218	0.305	0.752	0.517	98.041	0.943
219	0.045	0.294	0.196	24.246	0.974

Table S2. Parameters calculated from image analysis on 8x600

n	Area	Major	Minor	Angle	Circularity
1	0.592	1.041	0.724	36.218	0.955
2	0.435	1.069	0.518	17.828	0.813
3	0.562	0.932	0.768	45.774	0.985
4	0.577	0.947	0.776	37.157	0.992
5	1.945	1.574	1.573	90	1
6	0.776	1.036	0.954	101.997	1
7	0.315	0.645	0.622	90	0.994
8	0.633	1.078	0.747	29.741	0.951
9	0.662	0.953	0.884	49.911	0.999
10	0.499	0.832	0.763	59.645	0.995
11	0.214	0.545	0.499	39.607	1
12	0.087	0.346	0.319	48.592	1
13	0.17	0.488	0.445	82.644	1
14	0.457	0.791	0.735	86.383	1
15	0.253	0.707	0.455	53.139	0.92
16	0.461	0.773	0.76	0	0.998
17	0.65	1.121	0.738	41.354	0.934
18	0.161	0.611	0.335	6.64	0.886
19	0.069	0.398	0.221	10.315	0.918
20	0.243	0.644	0.481	82.699	0.968
21	0.957	1.407	0.866	54.651	0.916
22	0.397	0.896	0.564	27.615	0.923
23	1.248	1.423	1.117	35.361	0.976
24	0.772	0.994	0.988	45	1
25	0.193	0.499	0.493	31.751	0.978
26	0.716	1.11	0.822	41.566	0.972
27	0.135	0.484	0.355	22.305	0.954
28	0.173	0.542	0.406	45.002	0.965
29	0.073	0.353	0.263	35.872	0.973
30	0.393	0.817	0.613	171.684	0.981
31	0.419	0.734	0.727	134.835	0.996
32	0.726	0.965	0.958	100.575	0.996

33	0.57	0.989	0.735	100.817	0.965
34	0.132	0.462	0.365	168.737	0.977
35	0.489	0.791	0.788	0	0.989
36	0.687	1.034	0.845	36.658	0.981
37	0.58	0.859	0.859	50.807	0.989
38	0.527	0.999	0.672	25.545	0.946
39	0.132	0.462	0.365	13.353	1
40	0.328	0.734	0.57	159.07	0.969
41	0.493	0.9	0.698	79.825	0.977
42	1.388	1.513	1.168	49.803	0.975
43	0.08	0.345	0.294	174.031	0.947
44	0.053	0.293	0.229	76.76	0.947
45	0.189	0.559	0.431	79.441	0.974
46	0.554	1.119	0.631	13.691	0.885
47	1.042	1.532	0.866	31.453	0.884
48	1.953	1.992	1.249	34.565	0.92
49	0.58	0.966	0.764	51.34	0.981
50	2.129	1.893	1.432	42.227	0.968
51	0.093	0.397	0.299	128.736	0.969
52	0.741	1.123	0.84	39.647	0.966
53	0.159	0.51	0.398	161.611	1
54	0.372	0.799	0.592	36.439	0.987
55	0.107	0.422	0.322	42.122	0.946
56	0.214	0.601	0.452	62.661	0.974
57	0.12	0.447	0.343	42.312	1
58	0.428	0.857	0.636	35.536	0.968
59	0.184	0.525	0.446	96.688	0.992
60	0.081	0.347	0.298	24.176	1
61	0.173	0.506	0.436	43.14	0.987
62	0.17	0.493	0.44	77.626	0.984
63	0.199	0.554	0.457	8.09	0.976
64	0.735	1.123	0.834	31.206	0.968
65	0.752	1.239	0.772	47.774	0.912
66	0.554	1.066	0.662	51.23	0.928
67	0.114	0.483	0.299	140.279	0.909
68	0.122	0.493	0.314	117.608	0.948
69	0.069	0.374	0.235	138.789	0.899

Table S3. Parameters calculated from image analysis on 4x400

n	Area	Major	Minor	Angle	Circularity
1	0.493	1.158	0.542	165.208	0.816
2	0.307	1.007	0.388	2.673	0.727
3	0.255	0.682	0.477	172.45	0.963
4	0.232	0.702	0.421	146.912	0.915
5	0.191	0.566	0.429	130.577	0.966
6	0.231	0.626	0.471	1.679	0.971
7	0.203	0.617	0.419	166.763	0.944
8	0.322	1.096	0.374	163.12	0.67
9	0.276	0.645	0.546	168.353	0.986
10	0.083	0.358	0.297	158.499	0.996
11	0.236	0.595	0.505	156.034	0.992
12	0.14	0.452	0.395	147.891	1
13	0.112	0.406	0.35	145.551	1
14	0.642	1.612	0.508	167.14	0.639
15	0.272	0.781	0.443	146.57	0.898
16	0.05	0.329	0.195	127.067	0.886
17	0.114	0.411	0.352	124.553	0.981
18	0.193	0.665	0.369	151.388	0.884
19	0.071	0.398	0.227	163.004	0.901
20	0.112	0.5	0.284	111.442	0.882
21	0.104	0.482	0.275	165.799	0.891
22	0.121	0.519	0.296	169.636	0.884
23	0.074	0.308	0.308	0	1
24	0.593	1.386	0.545	171.817	0.737
25	0.323	1.019	0.403	174.819	0.747
26	0.224	0.691	0.413	167.173	0.908
27	0.118	0.503	0.298	160.295	0.896
28	0.067	0.377	0.228	61.264	0.896
29	0.058	0.349	0.213	165.269	0.93
30	0.072	0.39	0.235	146.239	0.903
31	0.081	0.41	0.253	167.847	0.921
32	0.116	0.497	0.296	3.005	0.917
33	0.649	1.315	0.628	171.095	0.823
34	0.38	1.007	0.48	168.168	0.824
35	0.138	0.608	0.289	163.914	0.814
36	0.226	0.776	0.371	151.943	0.813
37	0.162	0.659	0.313	162.417	0.818
38	0.107	0.529	0.257	146.416	0.819
39	0.467	1.539	0.386	172.494	0.539
40	0.472	1.228	0.489	164.999	0.743
41	0.1	0.564	0.225	155.813	0.749
42	0.268	0.799	0.428	162.281	0.869
43	0.076	0.334	0.291	149.43	0.969

44	0.077	0.335	0.292	158.52	1
45	0.123	0.439	0.356	164.825	0.975
46	0.256	0.644	0.507	149.881	0.982
47	0.795	1.413	0.717	169.06	0.846
48	0.771	1.1	0.892	141.519	0.983
49	0.087	0.409	0.271	157.88	0.937
50	0.224	0.658	0.434	155.847	0.935
51	0.395	0.885	0.568	153.299	0.929
52	0.597	1.446	0.526	153.519	0.695
53	0.681	1.54	0.563	166.787	0.702
54	0.534	1.245	0.547	170.9	0.78
55	0.201	0.763	0.335	159.356	0.78
56	0.098	0.527	0.236	157.517	0.782
57	0.282	0.899	0.4	145.038	0.79
58	0.06	0.408	0.187	142.965	0.784
59	0.067	0.394	0.218	127.278	0.867
60	0.024	0.235	0.131	127.027	0.841
61	0.045	0.239	0.239	0	1
62	0.035	0.21	0.21	0	0.897
63	0.167	0.571	0.372	145.992	0.943
64	0.168	0.568	0.377	164.754	0.904
65	1.109	2.104	0.671	168.208	0.643
66	0.076	0.358	0.27	122.114	0.982
67	0.124	0.459	0.343	152.575	0.987
68	0.351	0.897	0.499	167.076	0.882
69	0.576	1.516	0.484	171.387	0.642
70	0.041	0.297	0.175	166.5	0.894
71	0.528	1.236	0.544	168.495	0.788
72	0.069	0.296	0.296	0	1
73	0.346	1.208	0.365	174.691	0.613
74	0.617	1.624	0.484	172.792	0.611
75	0.293	1.009	0.369	159.05	0.707
76	0.088	0.446	0.253	163.171	0.885
77	0.179	0.749	0.304	162.501	0.746
78	0.219	0.746	0.374	152.286	0.833
79	0.135	0.435	0.396	112.168	1
80	0.098	0.414	0.3	167.12	0.97
81	0.072	0.382	0.24	9.917	0.914
82	0.144	0.545	0.336	152.316	0.9
83	0.157	0.52	0.384	158.949	0.956
84	0.064	0.305	0.269	119.636	0.989
85	0.061	0.299	0.261	125.768	0.963
86	0.076	0.334	0.291	120.57	0.966
87	0.053	0.276	0.245	115.285	1
88	0.058	0.289	0.257	136.802	1
89	0.076	0.331	0.294	136.916	0.983

90	0.055	0.293	0.241	134.997	1
91	0.044	0.264	0.211	134.998	1
92	0.159	0.527	0.385	167.067	0.967
